# 

**Published:** 1884-04

**Authors:** 


PAGE

ORIGINAL COMMUNICATIONS.—Eclampsia Parturientum. F. S. Billings, D.V.S. .......... 117
Scarlet Fever in Horses. John C. Peters, M.D. .......... 134
Abstract of the Investigations thus far made in the use of Equine Scarlatinal Virus. Joseph W. Stickler, M.D. .......... 145
Enteritis in an Elephant. George W. Bowler, M.D.V.S. .......... 149
Veterinary Sanitary Science and Preventive Medicine. Robert Ward, F.R.C.V.S. .......... 150
Neuroretinitis Optica .......... 153
Comparative Physiology of Menstruation. Alfred Wiltshire, M.D., F.R.C.P. .......... 163
EDITORIAL.—Veterinary Medicine in the United States—Foot and Mouth Disease—Latest Trichin-Epidemics in Germany—The Royal Veterinary School at Budapest, Hungary, as at present organized—Semi-annual Meeting of the United States Veterinary Association—Ninth Annual Commencement of the American Veterinary College—Correction—Suspension of Columbia Veterinary College—Fire at the New Veterinary College, Edinburgh—Dr. Salmon's Pro ective Virus Investigations—Wanted, A Dog Hospital .......... 172
CORRESPONDENCE .......... 193
CASE DEPARTMENT.—Haematorrhacis or Meningeal Apoplexia—Was it Glanders? .......... 195
REVIEWS .......... 198
PROGRESS OF VETERINARY SCIENCE.—The Cause of "Seedy Toe"—A Case of Cramp in a Cow—Bacteria in a Horse—Cancer in Domesticated Animals—Treatment of Glanders and Farcy by the Dosimetric System of Therapeutics .......... 206
OBITUARY .......... 211
NEWS AND MISCELLANY.—Inoculation of Dogs with Syphilis—A Cowman attacked by Foot and Mouth Disease—Scenes in Pasteur's Workshop—The Department of Veterinary Medicine in the University of Pennsylvania .......... 211

